# Revisiting the role of international developmental-behavioural paediatric fellowship training: a developing country’s perspective

**DOI:** 10.5116/ijme.6923.3f20

**Published:** 2025-11-29

**Authors:** Jun Jean Ong, Ranjini S. Sivanesom, Ashikin Mohd Nordin, Wee Vien Khoo, Gehan Roberts

**Affiliations:** 1 Department of Paediatrics, School of Medicine, IMU University, Malaysia; 2 Prince Court Medical Centre, Malaysia; 3 Department of Paediatrics, University of Cyberjaya, Faculty of Medicine, University of Cyberjaya, Malaysia; 4 Department of Paediatrics, Faculty of Medicine, University Malaya, Malaysia; 5 Centre for Community Child Health, Royal Children's Hospital, Australia

**Keywords:** International developmental-behavioural, Developmental Behavioural Paediatrics (DBP), paediatric fellowship training, paediatric training, Malaysia

## Introduction

International fellowships play a beneficial role in specialty medical training by providing opportunities for fellows to engage in the educational, professional, and cultural experiences of a different country.^1^ Fellows from countries with advanced healthcare systems enhance their skills through the experience of working with patients living with limited resources, with diseases less commonly encountered and of different cultural backgrounds.^2^ Similarly, fellows from low-middle-income countries undertaking fellowships in high-income countries reported significant advancement in their leadership and professional development leading to improvements in healthcare services and mentorship of future fellows in their home country.^3^ The fellowship fosters the sharing of knowledge and skills, inspires collaborations and work opportunities through the wide social networking made during the training. However, international fellowship training comes with its own set of challenges.  This article discusses the challenges, role and future position of international fellowships in Developmental-Behavioural Paediatrics (DBP) training in Malaysia. The discussion would add valuable insight into international fellowships programmes of subspecialty trainees from a less medically advantaged country, as reports from this region are scarce. The co-authors, comprising of prior trainees and training supervisors have contributed to its content.

### Developmental-Behavioural Paediatric Training in Malaysia

Developmental Behavioural Paediatrics (DBP) subspecialty is an expanding area of interest as developmental-behavioural conditions account for two-thirds of new and review consultations among general paediatricians.^4^ The growing demand for DBP expertise within Malaysia’s evolving healthcare system remains unmet with fewer than 20 developmental paediatricians currently registered in the national specialist register to serve a population of 33 million. DBP training in Malaysia is a structured three-year fellowship programme for recognised paediatricians who have completed their general paediatrics training. Selected trainees in subspecialty programs in Malaysia are often highly qualified, with more than 10 years of work experience after undergraduate training, as the limited specialty and subspecialty training positions are highly competitive.^5^ The program includes two years of work training at accredited local tertiary hospitals, in DBP specialty and other related medical and allied health specialties, followed by a year of an international fellowship program. The complex framework of DBP training is due to its’ close interaction with various socio-environmental, genetic and medical factors with child development and co-involvement with other conditions, necessitating interprofessional training in various medical, allied health and community settings.^6^  Trainees are expected to acquire competencies in various areas of development-behavioural paediatrics and disability through direct supervision and mentorship from professionals across different backgrounds.^7^ The training program equips trainees with the foundational skills required to establish future child development services and to pursue further professional development in their area of interest. Their progress is evaluated through continuous workplace-based assessments and their achievement in professional development activities and research work. Upon completion of the training program, trainees must submit their portfolio that meets the requirements of the National Specialist Register (NSR) before they can be certified by the training committee as a recognised developmental paediatrician.

International fellowships play a pivotal role in the training education model for most subspecialty training in Malaysia. During the post-independence era, extended international fellowships were necessary to address the shortage of expertise required to develop the local healthcare system.8 The return of fellows after completion of their training enables the development of local medical education institutions, followed by the establishment of various local subspecialty training programs. International fellowships were subsequently shortened to a one-year program to serve as an adjunct to the local subspecialty training. The overseas training period in a more developed country fosters valuable friendships, exposure to different healthcare models and advanced medical settings, and opportunities to participate in high-level research. Through international fellowships, trainees not only consolidate their core competencies but also gain expertise in their areas of interest and exposure to DBP practice domains less developed in their home country.

In recent years, the expansion of several other local subspecialty training programmes has rendered in international fellowships as an optional pathway. Although international fellowships remain obligatory for the completion of DBP training in Malaysia, the prospect of a similar change has been raised. The establishment of an alternative full local DBP training would raise more interest and offer more flexibility for future trainees, however, there are concerns about how this gradual paradigm shift would impact the quality of training. Considering the relatively young and small DBP fraternity in Malaysia, the concerns have fuelled discussion on whether a 3-year local training program can offer a comparable alternative training pathway.

### The Challenges

The current framework of DBP training programme is clearly defined. During the initial two years, fellows are trained in various multidisciplinary clinical rotations, participate in academic and community activities, while also fulfilling general paediatrics obligations at certified local public or university hospitals. Here, we focused on the specific challenges of international fellowships that commence in the third year of training.

### Lengthy Applications and Registration Processes

The preparation to enrol in international fellowships begins in the early phase of training. The first step involves securing a training position in the preferred recognised institution via an interview and where applicable, attain the required standards in an English proficiency test. Subsequent processes include acquiring numerous prerequisites to register as a practising medical doctor of the intended country, followed by a visa application. The entire process must be done promptly and in an orderly sequential method to avoid delays or losing the training position.

### High Financial Costs

International fellowships in high-income countries come with significant financial burdens for fellows travelling from low- and middle-income countries. The cost of various registrations and applications, living expenditures, selected course fees, health insurance and travel expenses are among the areas of expenditure necessary for fellows who enrol in the programme. Although financial loans may be provided, additional costs incurred are inevitable, particularly when accompanied by their families.

### Limited Oversea Training Centres

The increasing number of DBP fellows in training is a positive development. However, fellowship positions at previous renowned training centres worldwide have become highly competitive due to a large number of international applicants and resident trainees. Early applications and initiatives to explore new training sites are required to minimise the risk of an extension.

### Socio-Economic Disruptions and Uncertainties

Fellows enrolled are typically in the early to mid-stages of their careers and may have difficulties finding an adequate balance or solution to the substantial social changes that are expected during their fellowship. Many fellows are away from their families during both international and local training due to the urban locations and the limited number of training hospitals in Malaysia. After completion of training, fellows continue to be anxious about the uncertainties of their subsequent work placement site and the financial bond that comes with the training, limiting the flexibility of their socioeconomic position.

### Socio-cultural Adaptation and Application in the Local Context

Besides being away from family and friends, the endeavours of working in a new country during the initial months are expected. Fellows would need some time to familiarise themselves with the healthcare, education and related support systems, and adapt to the local colloquial language practices and cultural norms, especially in countries with large culturally and linguistically diverse populations. Although the time taken to adapt may seem to be a valuable character-building experience, the perceived usefulness in DBP training is unclear.

### Experiences of Past Fellows

The COVID-19 pandemic was an unexpected event that posed additional challenges to DBP training in the past few years. The heavy clinical workload and relocation of fellows to meet the urgent needs of the COVID-19 workforce have led to the extension of their training period. International fellowships were also initially interrupted, with Australia, among the first to welcome back international fellows from Malaysia. Despite the drawbacks, the travel restrictions imposed have presented a unique experience for fellows. The experiences with deserted airports, army escorts, prolonged travel quarantines and repeated viral testing were daunting but memorable. The changes to healthcare services implemented to conform with social restrictions have also presented fellows with various new practices, e.g., online teachings and telehealth that have persisted post-pandemic. The evolving changes over time warrant a discussion on the role of international fellowships in modern-day DBP training. The following informal narratives present the authors’ account of their experiences during training at an Australian tertiary hospital, with the hope of enriching reader’s insight into the strengths and challenges of the training.

#### Fellow A (Year 2021)

‘Year 2021 began well, with orientation, teachings and patient encounters fully conducted in-person during the first 5 months. The frequent physical gatherings were helpful in helping international fellows settle into the new working environment and connect with resident trainees and consultants. A series of lockdowns was enforced from May to October 2021, leading to drastic changes in clinical practice and daily routine. Most patient encounters were delivered through telehealth, while discussions and learning sessions were converted to online. The transition was slow in the beginning, but the demand to maintain quality in patient care led to the rapid improvement in our telehealth proficiency and communication skills. Despite efforts, some degree of discontinuity in clinical care was still experienced by patients due to the closure of intervention, educational and child support centres. On a positive note, fellows were inspired to approach problems creatively and seek opportunities within the present limitations. The increasing proficiency of digital device usage among clinicians and the community is a good example, which has made care team meetings with families and teachers more present and convenient. The opportunities for research, social networking and additional clinical observations may be less apparent but were certainly not diminished.'

#### Fellow B (Year 2022)

‘Year 2022 began slowly, with the majority of sessions and patient encounters conducted online due to the high numbers of COVID-19 positive cases. As the case numbers plateaued, in-person patient encounters were conducted more frequently. Approximately half remained as telehealth consultations particularly among follow-ups, patients living a distance away and unwell patients who required self-quarantine. Teaching sessions continued to be mostly online for practical reasons and to minimise the risk of contracting an infection. Problems with high screen time, such as poor focus, digital burnout and lack of informal social interaction continued to be a challenge. However, the easing of social restrictions gradually opened opportunities for out-of-hospital visits to places like Tweddle as well as other local community-supported organisations.’

#### Fellow C (Year 2023)

‘Healthcare practices in Year 2023 were mostly similar to the pre-COVID period, with most patient encounters conducted in-person. However, teaching sessions and supervision continued online, with many upgraded to a hybrid format that offered both onsite and online options that are flexible and favoured by all. The ease of using online communication devices among practitioners promoted its continuity post-COVID. Unfortunately, the frequency of physical social gatherings has not returned to the anticipated levels. The full reopening of schools and care centres provided an opportunity to engage with schools and to work closely with the community.’

The fellows’ narrative of their international fellowship training experience has been encouraging despite prior reports of the negative impact of COVID-19 on various training programmes.^9^ The challenges created opportunities for fellows to acquire new skills necessary to maintain the standard of care for patients, within the limitations of the pandemic. There was also no shortage of patients referred for DBP consultation, unlike that observed in other medical specialties.^9^ In addition, the availability of advanced telehealth services and an efficient workflow system facilitated the smooth transition to virtual consultations and continuity of outpatient services.^10^ Fellows acquired new competencies in telehealth communication, which will prove to be a valuable experience that will persist beyond the pandemic.^11^

Teaching/learning sessions conducted online or by hybrid mode continued to be organised regularly, with plenty of discussions, presentations, research and learning opportunities available. Strategies to foster an interactive environment during workshops were helpful, while supervisors were open to discussions on the possible deficiencies in training and additional learning opportunities. As expected, many experienced mental fatigue and reduced social connectedness due to the high digital usage and social isolation. Having said that, fellows enrolled in the international fellowships have had protected time on DBP output and research as they are less distracted by general paediatric duties and other public service disruptions back in their home country. The experiential learning has widened one’s perspectives and outlook on the possibilities of future healthcare service delivery back home.

### Moving forward

International fellowship complements local training programmes by providing added opportunities for professional growth and personal development. As reported by Streeton et al. (2021), overseas placement is an integral part of developing leadership skills through a better understanding of another health system and cultivating cultural intelligence that is important in working effectively with others.^12^ The inclusion of overseas postings can also be a strong motivation for fellows to excel.^13^ Despite the recognised advantages, the role of international fellowship as a requisite in DBP training in Malaysia is carefully evaluated due to various reasons. In addition to the discussed challenges, the advantages and impact of international fellowships in enhancing local training may be difficult to substantiate and often highly individualised. Comparative studies with control groups measuring the impact of international fellowship programs on future healthcare services in low-middle-income-countries and on professional development are limited as well. Therefore, it is understandable that many may perceive the benefits of international fellowship as not outweighing the challenges that come with the program. Additionally, the disruptions during the pandemic, the rise and success of digital engagement globally post pandemic, and the possibility of online educational networking raise possibilities of international collaborations without the need for overseas placements.^14^ However, given the limited evidence on the sustainability and outcomes of long-term online collaboration, particularly among new social networks, its usage should be explored with caution.

The international fellowship program addresses existing gaps and unmet needs of the initial two years of local training. The shortage of DBP practitioners and child development services, with a limited number of recognised training institutions, is among the significant training gaps that limit the exposure to the breadth and complexity of DBP practices. Extensive measures in expanding the DBP fraternity and services while strengthening collaborations between the stakeholders involved in DBP training are much required to facilitate the development of a full local DBP training programme. The experiences of returning fellows from various medically advanced countries will be instrumental in evaluating and accelerating the growth of the programme. Besides establishing the country’s DBP services, refining and expanding the training curriculum are necessary to ensure that training quality is preserved.^15^ Incorporating exciting collaborations with potential regional and international DBP institutions into the programme, through online coaching, electives and short-term placements, are opportunities to explore in creating a robust local DBP training. Recognising the wide and complex scope and needs of DBP practice, alternative opportunities during the third year of training to pursue personal interest in social research, local humanitarian or social justice sectors and government corporations responsible for developing policies should be offered to build a DBP fraternity with diverse skillsets.  Moving forward, a comprehensive three-year local DBP training programme with an optional international fellowship is an exciting prospect to look forward to in the future, however, much work is needed in establishing DBP services and addressing the gaps in training.  At present, international fellowships remain a crucial component of DBP training in Malaysia and continued support is warranted.

### Acknowledgement

We sincerely thank all professionals, both local and international, for their time and commitment to the training of DBP fellows.

### Conflict of Interest

The authors declare that they have no conflicts of interest.
